# An Integrated Systems Approach to Decode the Impact of Adolescent Nicotine Exposure in Utero and Postnatally Oxycodone Exposed Offspring

**DOI:** 10.21203/rs.3.rs-2753084/v1

**Published:** 2023-04-06

**Authors:** Adrian Flores, Austin Gowen, Victoria L. Schaal, Sneh Koul, Jordan B. Hernandez, Sowmya V. Yelamanchili, Gurudutt Pendyala

**Affiliations:** University of Nebraska Medical Center (UNMC); University of Nebraska Medical Center (UNMC); University of Nebraska Medical Center (UNMC); University of Nebraska Medical Center (UNMC); University of Nebraska Medical Center (UNMC); University of Nebraska Medical Center (UNMC); University of Nebraska Medical Center (UNMC)

**Keywords:** oxycodone, nicotine, behavior, adolescent, inflammation, synaptic biology

## Abstract

Perinatal exposure to prescription opioids pose a critical public health risk. Notably, research has found significant neurodevelopmental and behavioral deficits between *in utero* (IUO) and postnatal (PNO) oxycodone-exposed offspring but there is a notable gap in knowledge regarding the interaction of these groups to other drug exposure, particularly nicotine exposure. Nicotine’s widespread use represents a ubiquitous clinical interaction that current research does not address. Children often experiment with drugs and risky behavior; therefore, adolescence is a key timepoint to characterize. This study employed an integrated systems approach to investigate escalating nicotine exposure in adolescence and subsequent nicotine withdrawal in the IUO- and PNO-offspring. Western blot analysis found alterations of the blood-brain barrier (B.B.B.) and synaptic proteins. RT-qPCR further validated immune dysfunction in the central nervous system (CNS) consistent with compromised B.B.B. Peripheral nicotine metabolism was consistent with increased catabolism of nicotine concerning PNO & IUO, a predictor of greater addiction risk. Lastly, behavioral assays found subtle deficits to withdrawal in nociception and anxiety-like behavior. This study showed, for the first time, the vulnerabilities of PNO- and IUO-exposed groups concerning nicotine use during early adolescence and withdrawal.

## 1. Introduction

The opioid epidemic represents the advent of significant public health challenges, setting the stage for substantial public health cost (Seaton et al., 2007; Pouryahya et al., 2020). The most readily prescribed and abused opioids include buprenorphine, morphine, and oxycodone (OXY), each often prescribed to women as an analgesic for postpartum treatment (Hodder et al., 2021). OXY is teratogenic and readily crosses the placental barrier leading to impairments in fetal development (Pouryahya et al., 2020). Several of our previously published studies have provided evidence of phenotypic, behavioral, bio-chemical, and synaptic vulnerabilities of postnatal OXY-exposed offspring (PNO) and in utero OXY-exposed offspring (IUO) in early life and adulthood (Doberczak et al., 1991; Jones et al., 2010; Shahjin et al., 2019; Odegaard et al., 2020a; Odegaard et al., 2020b; Odegaard et al., 2022). Evidence of neuro-logical vulnerabilities identified in opioid-exposed offspring is further complicated when considering routinely abused substances (nicotine and alcohol). The interaction between perinatal OXY exposure and adolescent vulnerability is a significant gap in our knowledge.

Youth are frequently tempted with nicotine and alcohol in adolescence, a vulnerable timeframe. Adolescence (years 14–19) is a critical developmental window characterized by decreased inhibition, increased impulsivity, and the emergence of neuropsychiatric dis-orders (Caballero et al., 2016; Dwyer et al., 2019). At this age range development of the rodent forebrain follows a similar trajectory to human development, which undergoes considerable restructuring as it matures(Kopec et al., 2018). Nicotine use has been shown to disrupt this restructuring (Dwyer et al., 2019). Studies also provide evidence nicotine can disrupt the development of tertiary mechanisms for influencing impulsivity such as neurotransmitter re-uptake and metabolism (van der Put et al., 2014). Due to these vulnerabilities to nicotine in adolescence we hypothesize synaptic and behavioral out-comes will be exacerbated in these offspring.

Our integrated systems approach found phenotypic, molecular, and synaptic deficits in these offspring. Deficits were modest compared to known deficits animals experience. However, this study aimed to use a novel dose escalation of nicotine to mirror natural progression of nicotine habit forming. Therefore, results shown here are clinically relevant.

## 2. Materials And Methods

### 2.1 Animal Care

Sprague Dawley female rats were purchased from Charles River Laboratories Inc. (Wilmington, MA, U.S.A.). Females purchased as virgins (IUO) or 19 days pregnant (PNO). The animals are housed in a 12-hour light-dark cycle and fed ad libtum. All procedures and protocols were approved by the Institutional Animal Care and Use Committee of the University of Nebraska Medical Center (IACUC) and conducted following the National Institutes of Health Guide for the Care and Use of Laboratory Animals.

#### 2.1.1 Oxycodone and nicotine treatment

**Figure 1:**
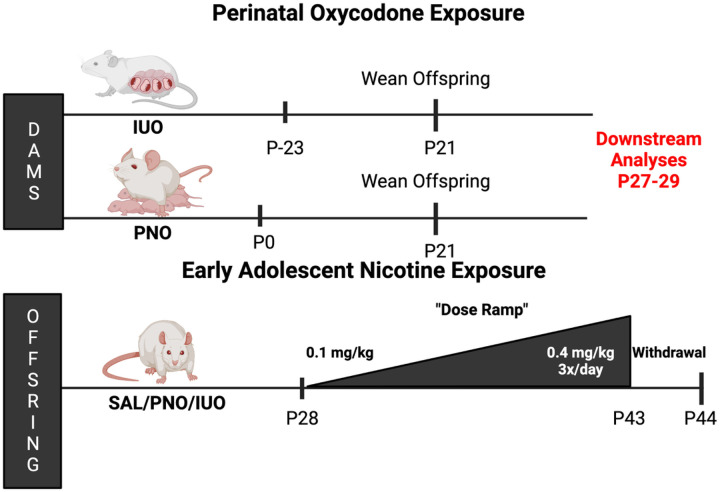
Nicotine and oxycodone administration paradigm. This paradigm details the nicotine administration timing, dose escalation, withdrawal condition used and termination. Generated using Biorender.com

The treatment paradigm was adapted from our labs’ previous preclinical animal model to include nicotine hydrogen tartrate salt (Sigma Aldrich, Milwaukee, WI, U.S.A.) exposure during early adolescence (P28–43) of IUO & PNO groups [6, 7, 9, 15]. The IUO animals were orally gavaged with 15 mg/kg/day (chronic oxycodone dosage) oxycodone HCl (Sigma Aldrich, St. Louis, MO, U.S.A.) dissolved in saline vehicle. PNO groups were gavaged starting at P0, and IUO groups were gavaged starting one week before mating with a proven breeder. The IUO group was dosed throughout the pregnancy and both groups continued drug treatment until weening. Nicotine treatment followed a dose escalation model to reflect more natural progression of habit formation. However, these doses are considered appropriate because of the gestational vulnerabilities mentioned. Nicotine hydrogen tartrate salt dissolved in saline vehicle was administered subcutaneously (S.C.) using the following escalation paradigm, 1.) initial injections of 0.1 mg/kg/day on days P28–29, 2.) 0.2 mg/kg/day injection days P30–31, 3.) 0.4 mg/kg/day days P32–33 and 4.) 0.4 mg/kg 3 times a day for the remainder of the drug treatment (P32–43). Single day dosing was performed at 9:00 AM; dosing three times a day was performed at 9:00 AM, 12:00 PM, 3:00 PM. The nicotine regimen is represented in Fig. 1. All animals were monitored daily. At P43 tissue was harvested and stored at −80°C until subsequent analyses. Animals that underwent a 24-hour withdrawal period were sacrificed on P44.

### 2.2 Biochemical studies

#### 2.2.1 Liver protein isolation

Liver tissue was homogenized with T-PER Lysis Buffer (Thermo Fischer Scientific) containing protease/phosphatase inhibitors. Samples were spun supernatant was collected. Pierce B.C.A. protein assay kit (Thermo Fischer Scientific) was used to determine protein concentration for western blot analysis.

#### 2.2.2 Brain lysates and synaptosomes

Brain tissue from the frontal cortex was homogenized with 10x volume of lysis buffer containing protease/phosphatase inhibitors and spun at 0.3 × g for 5 minutes. The super-natant was collected, and 100 μl aliquot (brain lysate) was collected for western blotting of blood-brain barrier proteins. The supernatant was spun at 12,000 × g for 20 minutes, dis-carded, and the remaining pellet was resuspended in 200 μl of 1X PBS containing protease/phosphatase inhibitors. Pierce B.C.A. protein assay kit (Thermo Fischer Scientific) was used to quantify and prepare samples for further analysis.

#### 2.2.3 Western blot

Western blot was adopted from studies performed by Odegaard and colleagues (Odegaard et al., 2020a). Brain and liver lysates, and synaptosomes from each animal were loaded into SDS PAGE 10 well gels (Invitrogen, Waltham, MA, USA) under reducing conditions, followed by transfer to a nitrocellulose membrane using iBlot2 (Invitrogen) and immunodetection. Concentrated Superblock was used to block nonspecific binding (Thermo Fisher Scientific, Waltham, MA, USA). After blocking, membranes were incubated overnight at 4°C with a primary antibody. Primary and secondary antibody dilutions were done according to the manufacturer’s suggestion and are shown inTable 1.. Blots were developed using Azure cSeries Imager (Azure Biosystems, Dublin, CA, USA) with Super-Signal West Pico Chemiluminescent Substrate (Thermo Fischer Scientific).

#### 2.2.4 Cotinine ELISA

Serum cotinine level was measured using Cotinine ELISA Kit (cat. no. KA0930; Abnova; Taipei, Taiwan) according to the manufacturer’s guidelines and as previously described (Gupta et al., 2019). ELISA was then analyzed using Epoch System. Values were determined using the interpolated best fit (P4 curve).

### 2.3 Molecular studies

#### 2.3.1 RNA Isolation and Quantitative real-time PCR

A modified isolation procedure was performed, excluding prewash steps. Total RNA isolation of the prefrontal region was completed using Direct-zol RNA kits (Zymo Re-search Corp., Irvine, CA, U.S.A.). Monarch^®^ RNA Cleanup Kit (50 μg) (BioLabs, Ipswich, MA, U.S.A.) was used to improve purity of samples that did not qualify the 260/280 nm ratio of < 1.8. cDNA synthesis was performed with SuperScript IV Reverse Transcriptase kit (Invitrogen). Quantitative real-time PCR was performed using QuantStudio 7 System (Applied Biosystems) and evaluated neuroinflammatory and microglia activation. The inflammatory array included: allograft inflammatory factor-1 (Aif-1) analogous to Ionized calcium-binding adapter molecule-1 (IBA-1) in humans, interleukin-1 beta (IL-1β), inter-leukin-6 (IL-6), and tumor necrosis factor-alpha TNF-α. ΔΔCt was calculated to determine fold-change, and Graph Pad Prism was used to determine statistical significance (Odegaard et al., 2020a).

#### 2.3.2 Histology

Protocols used were adopted from Yang et al (Yang et al., 2016). For immunohistochemistry, sections (10 μm thick) from paraffin-embedded whole brains were deparaffinized and re-hydrated. Immunostaining with GFAP and IBA-1 antibodies involved boiling the tissue sections in citrate buffer (pH 7) for about 8 minutes for efficient antigen retrieval. Sections were blocked with 10% normal goat serum (NGS) for 2 h at RT and then incubated in primary anti-GFAP (1:500), or anti-IBA1 (1:250), antibody at 4°C overnight. The sections were then incubated with immunofluorescent secondary antibody at a 1:1000 dilution and DAPI (1:333) for 2 h. GFAP is shown in red. IBA-1 shown in green. Histological counting was performed in the ImageJ software. Figures represent hand counting.

### 2.4 Behavioral Assessments

#### 2.4.1 Marble burying

Marble burying was performed as described in our previous studies (Odegaard et al., 2020b). Testing was performed at P43. Animals that underwent a 24-hour withdrawal period were analyzed at P44. A rat cage (929 cm2, 43.18 × 21.59 × 20.32 cm) contained a leveled 5 cm layer of ¼-inch corncob bedding (Envigo #7097), and 20 standard glass marbles (15 mm diameter, 5.2 g) were lightly placed in a 5 × 4 arrangement along the bedding. The subject was placed into the cage, and the cage was covered for 30 min. The animal was removed, and the marbles were imaged and scored by a scorer blinded to the conditions. A marble was considered buried if more than 2/3 of a marble was under the bedding.

#### 2.4.2 Hot plate

Testing was performed P43–44. Animals that underwent a 24-hour withdrawal peri-od were analyzed at P44. Each animal was placed on an Incremental Hot Plate set at 25°C (IITC Life Science, Irving, CA, U.S.A.). Temperature increased at a rate of 5°C per minute with maximum temperature of 50°C. During testing animals were constantly monitored. When the animal licked its back paw, a standardized behavior to avoid heat, the test was stopped. Duration and intensity of heat was recorded using software provided by IITC Life Science and recorded by hand. The testing plate was sanitized between each animal to prevent litter mate scent from interfering with performance.

### 2.5 Data and statistical analysis

All data presented reported mean as ± S.E.M. All data in each analysis was normally distributed. Significance was determined using two-way ANOVA approach followed by Tukey’s test or Dunnett’s correction when appropriate with a significance criterion of p ≤ .05. Graph Pad.

## 3. Results

### 3.1 Phenotypic and metabolic vulnerabilities

**Figure 2:**
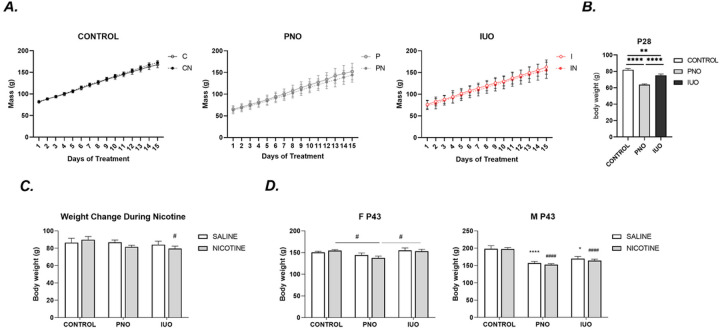
Body weight analysis. A. Mass of animals during nicotine treatment (P28-P43) n=37–60. B. Average weight gain during nicotine treatment. IUO-nicotine group displayed decreased weight gain during nicotine treatment when compared to IUO-sham. Two-way ANOVA followed by Tukey’s test: p-value: ≤.05 Error bars represent SEM: # ≤.05 when compared to sham counterpart. C-D. Weight gain observed during nicotine treatment is sexually dimorphic and significantly affected by the addition of nicotine. n=16–34. Two-way ANOVA followed by Tukey’s test: p-value: ≤.05 Error bars represent S.E.M. Represent significance when compared to Saline-counterpart: **** ≤.00001. # Represents significance when compared to PNO-counterpart: #### ≤.00001. S: Saline, SN: Saline-nicotine, PS: Postnatal oxycodone-saline, P.N.: Postnatal oxycodone-nicotine, I.S.: In utero oxycodone-saline, and IN: In utero oxycodone-nicotine. n=6–16.

PNO and IUO weights trended lower than controls as shown in Fig. 2. Weight re-ductions at P28 show a significant influence of perinatal OXY exposure (Fig. 2b.). P43 overall weight reductions demonstrate the impact of nicotine to exacerbate trends observed from OXY exposure. When observing the sex-based breakdown, PNO-groups over-all demonstrated the most difficulty gaining weight under nicotine treatment remaining lower in weight throughout the entire treatment regimen. Significantly impaired ability to gain weight was most notably displayed in males with lower weight observed in all conditions Fig. 2d. These findings suggest a weight-based vulnerability with opioid use and an increased susceptibility for males also using nicotine. The latter finding also suggests a potential hormonal element, as males tend to have slower and longer growth patterns.

**Figure 3:**
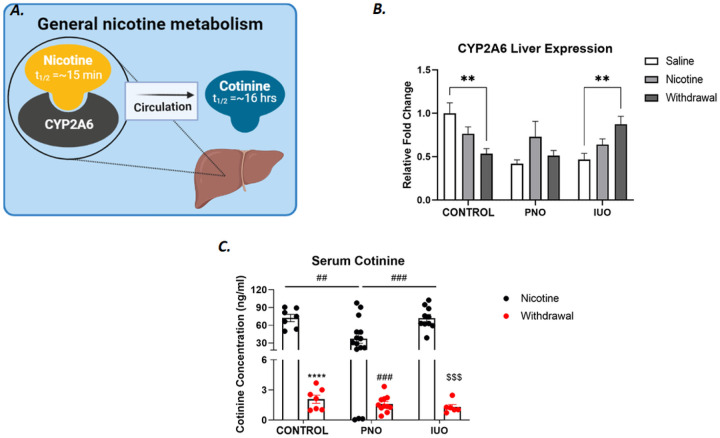
Enzymatic evaluation of CYP2A6. A. General overview of the rate-limiting step in nicotine metabolism. B. Western blot analysis on CYP2A6 expression experimental groups revealed the greater potential for metabolism nicotine in PNO and IUO groups. IUO observed an inverse stepwise trend as compared to saline expression of CYP2A6. C. PNO animals displayed greater nicotine metabolism following treatment. Saline-, PNO-, and IUO -withdrawal groups displayed a stepwise trend toward lower serum cotinine. Two-way ANOVA followed by Tukey’s test: p-value: ≤.05 Error bars represent SEM: **(##) ≤ .001, ### ≤.001 N=6/group/treatment. Created with Bio-Render.com

We next assessed the levels of cotinine, a principal metabolite of nicotine in the liver samples from the different treatments that showed unique regulation of nicotine metabolism (Fig. 3). PNO offspring trended higher in enzyme level. While IUO-offspring showed significant increases in enzyme level. Next, we used ELISA to assess the level cotinine in the serum. We found PNO offspring had significantly lowered cotinine in their serum than saline offspring at the end of nicotine treatment.

### 3.2 Blood-brain-barrier integrity

**Figure 4:**
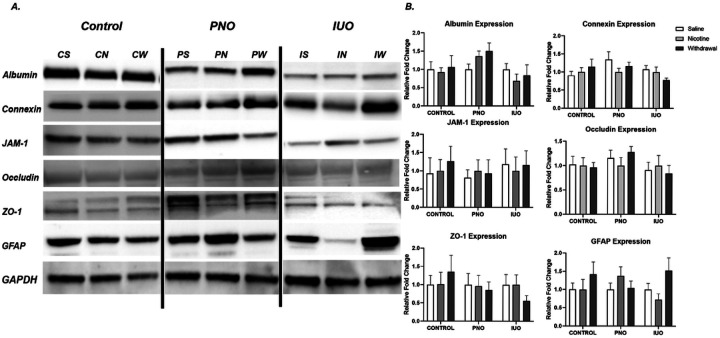
Blood-brain barrier expression analysis. Western blot analysis on homogenate samples isolated from PNO, IUO and saline showing possible compensatory regulation of junctional proteins. Two-way ANOVA followed by Tukey’s test when appropriate: p-value: ≤ .05. Error bars represent SEM: *≤.05. N=6/group/treatment.

We next looked at the BBB, which tightly regulates the peripheral and central nervous (CNS) interactions and is often altered in neurodegenerative disorders. As seen in Fig. 4, these proteins were subtly downregulated in western blot analysis. Non-disruptive alterations such as those depicted suggest a subtle change in barrier permeability. Subtle changes in permeability can give rise to inflammatory phenotypes in the brain and changes in function at the synapse level that are difficult to evaluate.

### 3.3. Synaptic alterations

**Figure 5:**
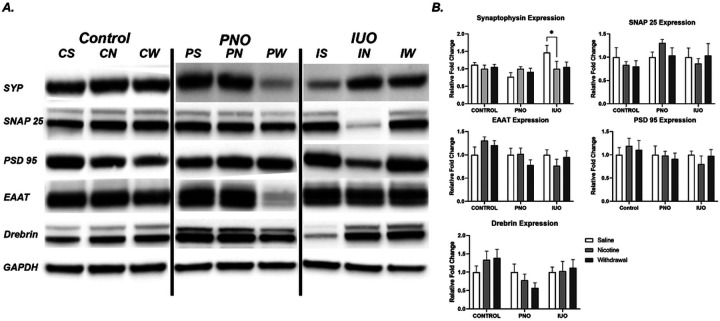
Synaptic protein and neurotransmission analysis. Western blot analysis on synaptosomal samples isolated from PNO, IUO, and saline demonstrating unique regulation. Synaptophysin is upregulated in IUO-withdrawal group. Two-way ANOVA followed by Tukey’s test when appropriate: p-value: ≤ .05. Error bars represent SEM: *≤.05. N=6/group/treatment.

To further understand the molecular impact of BBB dysregulation we analyzed crude synaptosomes isolated from the cortex for essential synaptic proteins associated with synaptic transmission and synaptogenesis (Fig. 5). Of the proteins analyzed, all were expressed with distinct patterns in response to treatment. Most remarkably, synaptophysin was downregulated in IUO-withdrawal and nicotine groups when compared to IUO-sham, indicating potential transmission challenges because of synaptophysin’s role in vesicle formation and over reactivity in withdrawal. Interestingly, saline animals experience an upward trend of excitatory amino acid transporter (EAAT) across nicotine and withdrawal while IUO-groups observed an inverse response. However, PNO demonstrates the opposite trend beginning high expression in control (PNO-sham) and low ex-pression in nicotine with even lower expression during withdrawal. IUO displayed di-minished downward trends suggesting the regulation of excess of excitatory neurotransmitters is diminished. Moreover, the up- and down-regulation of proteins associated with synaptogenesis, vesicular transport (SNARE complex), and excitatory signaling indicate vulnerabilities in adolescence and could persist into adulthood with unknown sequela.

### 3.4 Neuroimmune evaluation

**Figure 6:**
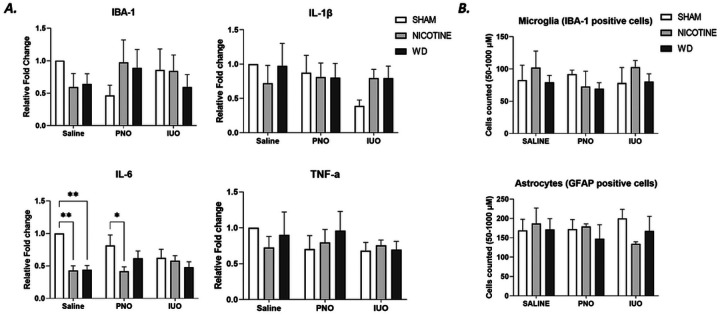
Cytokine production and microglia analysis. Interluekin-6 was significantly downregulated in nicotinetreated and withdrawal groups, with a less pronounced effect between PNO and IUO. Two-way ANOVA followed by Dunnett’s test: p-value ≤.05. Error bars represent SEM: *≤.05, ** ≤.001; IBA-1 N=4; IL-6, IL-1β, TNF-α N=5. B. Astrocyte and microglia counting. No significant differences were found. Two-way ANOVA followed by Tukey’s test when appropriate: p-value: ≤.05 Error bars represent SEM: *≤.05, ** ≤.001, n=5–6/group/treatment.

RT-qPCR was employed to monitor the inflammatory milieu and immune response found in our other studies. Interleukin-6 (IL-6) was significantly downregulated between nicotine use and sham Saline- and PNO-animals, as shown in Fig. 6. A sudden de-crease in IL-6 suggests a diminished pro-inflammatory response which literature characterizes as neuroprotective property of nicotine treatment (Imai et al., 1996; Stevens et al., 2003; Chen et al., 2013; Pittenger et al., 2018a; Rojas-Rodríguez et al., 2020).

Next, IBA-1 was used to characterize microglia. IBA-1 transcription was lower in saline nicotine animals when compared to saline sham animals. IUO-withdrawal animals corroborated the trend toward lower IBA-1 transcription. PNO-sham animals showed di-minished IBA-1 in opposition to IUO and Saline shams. IUO sham displayed diminished IBA-1. However, IHC staining shown in summarized in Fig. 6. did not corroborate trends in microglia or astrocyte. These findings provide some evidence that PNO and IUO react with nonclassical immune responses suggesting nicotine and withdrawal elicit an altered microglia activation and inflammatory response.

### 3.5 Behavioral deficits

PCR demonstrated that nicotine treatment in Saline- and PNO-offspring had lower inflammatory potential that interestingly did not occur in IUO-offspring. Analgesic and anxiogenic properties are defining features of opioids and nicotine use. These distinct inflammatory signatures and general knowledge of nicotine and OXY exposure lead us to hypothesize that under stress these PNO- and IUO-offspring exposed to nicotine in early adolescence will have distinct reactions. To address this hypothesis, we used the marble bury assay and hot plate assay.

**Figure 7:**
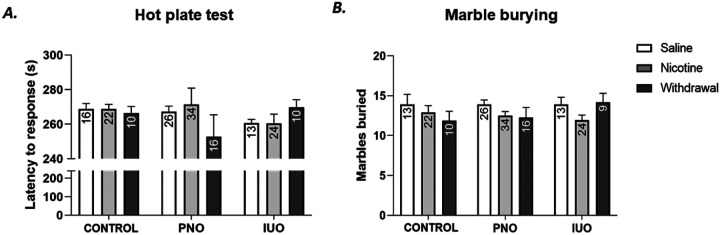
Behavioral analysis. A. Hot Plate analysis at P43 or P44. Overall, no significant differences were observed. PNO-withdrawal animals’ trend toward lower pain threshold when compared to PNO-saline. B. Marble burying assay revealed no significant difference in anxiety-like behavior between groups. Two-way ANOVA followed by Tukey’s test: p-value: ≤.05 Error bars represent S.E.M. n-value displayed in graphs.

Nicotine regulation and diminished cotinine levels in OXY-exposed groups suggest enhanced anxiety-liked behavior (compulsive-like) and lowered pain sensitivity. Accordingly, we used marbles buried to show anxiety level and delay to nocifensive response as an indicator pain threshold. Modest anxiety-like behaviors were observed in all animals, as shown in Fig. 7, inconsistent with the literature. This trend was not found in IUO groups. Hot plate testing revealed no significant difference between groups, as shown in Fig. 7. These findings suggest distinct nociceptive regulation of PNO and IUO groups during withdrawal and nicotine use. Furthermore, hot plate tested nociception in the dorsal root ganglion and demonstrated hot stimulus does not produce a significant difference in animal reaction.

## 4. Discussion

We employed a novel comparative analysis of vulnerabilities in PNO & IUO exposed off-spring to nicotine use in early adolescence using an integrated systems approach to evaluate molecular, enzymatic, synaptic, and behavioral deficits. We also monitored the im-mediate withdrawal of animals as this is an understudied and dynamic stress. Deficits corroborate our previous research elucidating baseline deficits of PNO- and IUO-offspring (Odegaard et al., 2020b).

Current literature establishes nicotine’s role in creating an ischemic brain environment and promoting microvasculature dysregulation in the PNS and CNS. (Babb et al., 2010; Koizumi et al., 2019). Western blotting was used to detect gap junction and tight junction proteins to elucidate B.B.B. integrity as they are impacted in acute and chronic addiction models (Hammad et al., 2019; Buzhdygan et al., 2021). B.B.B. analysis indicated dysregulation of gap junctions including an association between Al-bumin and Connexin expression. Connexin is the primary determinant of hemichannel stability. Hemi-channel activity is linked to the presence of neurotoxic metabolites, including inflammatory and degenerative signaling molecules (Gonçalves et al., 2017; Diaz et al., 2019). An increased presence of albumin through the B.B.B. could represent leakage. Strongly opposing trends of adhesion and junctional molecules, such as those observed in Fig. 4., could indicate novel regulatory mechanisms or impaired regulation of B.B.B. permeability, potentially promoting a “leaky” brain environment in the case of PNO or an ischemic brain in IUO groups. Gap junctions and tight junctions contribute to the function of several other GPCR’s and leukocyte migration. Dysregulation potentially sets the stage for further impacted neuroimmune environments in PNO- and IUO-offspring exposed to nicotine. Non-disruptive B.B.B. alterations such as those observed can also set the stage for altered inflammatory responses.

While RT-qPCR showed significant change concerning IL-6 production, an in-depth analysis of leukocyte activity, using flow cytometry on the spleen to investigate cell specific changes, could enhance our understanding of the observed trends. While IUO-sham animals showed lower nonsignificant endogenous production of IL-1b and IL-6 collectively, these results suggest IUO animals have lower capacity to mount an immune response. The typical neuroprotective effects of nicotine downregulate TNF-α and IL-10 and upregulate anti-inflammatory signaling. While TNF-α was not shown to be downregulated in these studies, evaluation of IL-10 and anti-inflammatory molecules (ar-ginase-1 or arginase-2) would help characterize the immune environment of OXY-exposed offspring.

Enzymatic analysis of the liver found that PNO and IUO displayed enhanced nicotine metabolism through induction of C.Y.P. Current opioid users have also demonstrated similar nicotine metabolism (Chen et al., 2020). PNO-nicotine animals expressed higher C.Y.P. activity relative to PNO-sham. A potential cause is that of cytochromes contains numerous polymorphisms, which could result in considerably more efficient drug metabolism (Hammad et al., 2019). In tandem, other studies using a Sprague Dawley rat model have investigated the role of nicotine in energy consumption and determined a direct impact on adipose deposition via the κ opioid receptor (KOR) by way of uncoupling protein 1 (UCP1) and altered mitochondrial function (Kumar et al., 2021). These studies offer a theoretical framework for our weight-based findings. The involvement of mitochondrial elements also suggests changes in reactive oxygen species (R.O.S.) production, which could contribute to neurochemical changes in the brain, such as our previous results in adult PNO and IUO [20]. Other stress models have found evidence of increased R.O.S. production contributing to neurochemical alterations. CYP-450-cytochromes produce high R.O.S. as a byproduct of drug metabolism and are commonly used as a biomarker of oxidative stress (Ande et al., 2012). Studies performed in vitro found C.Y.P. metabolism of nicotine can produce astrocyte cytotoxicity partially through R.O.S. induction (Ande et al., 2012). Common aberrations involving CYP-450 proteins and R.O.S. environment could also indicate a significant role in ferroptosis, a rapidly expanding avenue of addiction biology. Induced ferroptosis could contribute to neurochemical differences observed in the hippocampus previously published by this lab by increasing lipid peroxidation and decreasing cell integrity (Wikoff et al., 2008).

Elevated excitatory neurotransmitters are a feature of nicotine use and an ischemic brain environment like those seen in our B.B.B. analysis. Prior studies found lower AM-PA/NMDA receptor ratio and significantly increased glutamate in PNO and IUO (Subramanian et al., 2019; Odegaard et al., 2020b). EAAT alterations in nicotine-exposed animals suggest an enhanced role of excitatory neurotransmitters, possibly indicating structural changes in the astrocyte-neuron junction. Downregulation of scaffolding protein, Drebrin in IUO and PNO animals is also consistent with a more profound structural impact, such as ramified astrocytes and spine loss (Subramanian et al., 2019; Runge et al., 2020). Synaptophysin regulation in IUO also suggests impaired post-synaptic transmission and altered vesicle cargo (Gordon et al., 2011). Also, synaptophysin regulation in IUO also suggest impaired post-synaptic transmission and altered vesicle cargo (Gordon et al., 2011).

Proinflammatory cytokines were mostly downregulated in saline and PNO, which recapitulates the neuroprotective potential of nicotine. However, IL-6 can be pro- and anti-inflammatory, depending on the activity of other biomolecules such as a disintegrin and metalloproteinase domain 10 (ADAM10), indicating potential differences in the down-stream inflammatory response and neuroimmune changes. IUO’s lack of IL-6 induction may indicate that the neuroprotective effects of nicotine are ameliorated because of mostly diminished innate immune function, possibly due to chronic perinatal exposure to opioids. In more typical nicotine models the anti-inflammatory effects extend to other cytokines and chemokines. Mechanistic evaluation of nicotine in the context of IUO and PNO would help clarify changes in immune responses and how they contribute to the progression of neuropsychiatric disorders. This change also suggests that the pharmacokinetics of nAChR activity is significantly altered in IUO, in line with the altered glutamate activity observed. As such, IUO is potentially vulnerable to numerous neurological diseases and could necessitate using novel therapeutic strategies to account for unique nAChR behavior.

While OXY and nicotine use distinct mechanisms to provide desired effects, both can be used to ease various types of pain (Bruijnzeel, 2017; Pinho-Ribeiro et al., 2017; Gao et al., 2020). These studies observed minor differences associated with nociception (hot plate) and anxiety-like behavior (Fig. 7). Marble burying results indicate increased anxiety-like behavior in PNO & IUO offspring when using nicotine withdrawal and ameliorated anxiety-like behaviors. An elevated plus maze may demonstrate more significant anxiety-like behaviors as a mouse study recently demonstrated in a perinatal buprenorphine drug regiment (Martin et al., 2021). PNO demonstrated the most behavioral deficits suggesting an acute OXY treatment promotes more deficits. Von Frey testing done on IUO and PNO naïve animals saw more significant reductions in nociception in PNO and IUO animals [8]. Together these results suggest PNO and IUO demonstrate higher vulnerability to nociception concerning a mechanical stimulus versus thermal stimulation. Overall, we demonstrate behavioral deficits regarding nicotine and withdrawal. Nicotine use is known to alter neuropeptides such as neurotensin which is responsible for animal behavioral changes (Alburges et al., 2007; Bruijnzeel, 2017; Pittenger et al., 2018b). However, most studies used higher nicotine concentrations in their treatment plans, 2.0 mg/kg/day via S.C., intraperitoneal, or osmotic pump. Prospective studies could increase nicotine use in treatment as PNO, and IUO only experienced subtle behavioral changes during this low dose study. Using greater nicotine dosage in adolescence could pose more robust behavioral deficits and highlight molecular and synaptic alterations discussed. However, further escalating the nicotine dose in adolescence would put these vulnerable populations at risk for more severe health effects. Doing so would also lose the clinical relevance of our low dose ramp. While the behaviors observed provide the basis for investigating a broader impact, sex-based observations would provide robust results given the rich behavioral literature of opioid-exposed animals and nicotine-exposed animals.

Deficits corroborate our previous research elucidating baseline deficits of PNO- and IUO-offspring (Odegaard et al., 2020a; Odegaard et al., 2020b). Further mechanistic studies are needed to understand specific pathologies of nicotine-exposed PNO & IUO offspring. Data generated here also warrants the investigation of PNO & IUO interaction with other commonly abused substances such as alcohol. Further investigation of withdrawal because of the opposing inflammatory signature shown, B.B.B. permeability, behavior, and synaptic proteins. Altered synaptic proteomes have been observed in opioid and nicotine use, as such high-throughput approaches would provide novel data sets for robust analyses (Che and Roth, 2018; Gao et al., 2020). Results found pre-sented here provide the basis for further focused studies.

## 5. Conclusions

Collectively, our study using an integrated systems approach shows a comparative analysis of alterations in enzymatic, synaptic, molecular, and behavioral changes in OXY off-spring exposed to nicotine in adolescence. Findings establish that acute and chronic OXY promote unique neurological compensatory mechanisms regarding nicotine exposure in early adolescence. The full impact of these changes could persist into adulthood and compromise the longevity of dual exposed offspring.

## Figures and Tables

**Table 1.** Antibody inventory and details used in western blot analysis.

#	PROTEIN	1°	2°	REACTIVITY	LOAD	SOURCE	CAT#
**1**	Albumin	1:1,000	1:2,500	Mouse	10 μg	Santa Cruz Biotech.	sc-271605
**2**	GFAP	1:1,000	1:5,000	Mouse	10 ug	Sigma Aldrich	G3893-.2ML
**3**	Connexin-43	1:1,000	1:2,500	Rabbit	10 μg	Sigma Aldrich	C6219
**4**	CYP2A6	1:2,500	1:5,000	Mouse	10 μg	Invitrogen	MAS-25758
**5**	Drebrin	1:1.000	1:500	Mouse	10 μg	MBL	D029-3
**6**	EAATs	1:2.500	1:5.000	Rabbit	10 μg	Abeam	ab41621
**7**	GAPDH	1:2,500	1:5,000	Mouse	10 μg	Invitrogen	MAS-15738
**8**	JAM-1	1:1.000	1:2.500	Rabbit	10 μg	Abeam	ab125836
**9**	Occludin	1:500	1:1.500	Rabbit	30 μg	Invitrogen	71-1500
**10**	PSD95	1:2.000	1:5.000	Mouse	10 μg	Invitrogen	MA1-045
**11**	SNAP 25	1:2,000	1:5,000	Mouse	10 μg	Sigma Aldrich	S96S4
**12**	Synaptophysin	1:5.000	1:10.000	Rabbit	10 μg	Invitrogen	PA1-1043
**13**	ZO-1	1:1,000	1:2,500	Rabbit	10 μg	Invitrogen	61-7300

## Data Availability

The data that support the findings of this study are contained within the article.

## References

[1] Alburges ME, Hoonakker AJ, Hanson GR (2007) Nicotinic and dopamine D2 receptors mediate nicotine-induced changes in ventral tegmental area neurotensin system. Eur J Pharmacol 573:124–132. 17689525 10.1016/j.ejphar.2007.06.063 PMC2707996

[2] Ande A, Earla R, Jin M, Silverstein PS, Mitra AK, Kumar A, Kumar S (2012) An LC-MS/MS method for concurrent determination of nicotine metabolites and the role of CYP2A6 in nicotine metabolite-mediated oxidative stress in SVGA astrocytes. Drug Alcohol Depend 125:49–59. 22498344 10.1016/j.drugalcdep.2012.03.015 PMC3413753

[3] Babb M, Koren G, Einarson A (2010) Treating pain during pregnancy. Can Fam Physician 56:25–27. 20090076 PMC2809170

[4] Bruijnzeel AW (2017) Neuropeptide systems and new treatments for nicotine addiction. Psychopharmacology (Berl) 234:1419–1437. 28028605 10.1007/s00213-016-4513-5 PMC5420481

[5] Buzhdygan TP, Rodrigues CR, McGary HM, Khan JA, Andrews AM, Rawls SM, Ramirez SH (2021) The psychoactive drug of abuse mephedrone differentially disrupts blood-brain barrier properties. J Neuroinflammation 18:63. 33648543 10.1186/s12974-021-02116-z PMC7923670

[6] Caballero A, Granberg R, Tseng KY (2016) Mechanisms contributing to prefrontal cortex maturation during adolescence. Neuroscience & Biobehavioral Reviews 70:4–12. 27235076 10.1016/j.neubiorev.2016.05.013 PMC5074870

[7] Che T, Roth BL (2018) Phosphoproteomics Illuminates Opioid Actions. Biochemistry 57:5505–5506. 30179451 10.1021/acs.biochem.8b00809

[8] Chen Y, Nie H, Tian L, Tong L, Yang L, Lao N, Dong H, Sang H, Xiong L (2013) Nicotine-induced neuroprotection against ischemic injury involves activation of endocannabinoid system in rats. Neurochem Res 38:364–370. 23192660 10.1007/s11064-012-0927-6

[9] Chen YC, Fowler JP, Wang J, Watson CJW, Sherafat Y, Staben A, Lazarus P, Denton TT, Fowler CD (2020) The Novel CYP2A6 Inhibitor, DLCI-1, Decreases Nicotine Self-Administration in Mice. J Pharmacol Exp Ther 372:21–29. 31628204 10.1124/jpet.119.260653 PMC6904882

[10] Diaz EF, Labra VC, Alvear TF, Mellado LA, Inostroza CA, Oyarzun JE, Salgado N, Quintanilla RA, Orellana JA (2019) Connexin 43 hemichannels and pannexin-1 channels contribute to the alpha-synuclein-induced dysfunction and death of astrocytes. Glia 67:1598–1619. 31033038 10.1002/glia.23631

[11] Doberczak TM, Kandall SR, Wilets I (1991) Neonatal opiate abstinence syndrome in term and preterm infants. J Pediatr 118:933–937. 2040931 10.1016/s0022-3476(05)82214-0

[12] Dwyer JB, Cardenas A, Franke RM, Chen Y, Bai Y, Belluzzi JD, Lotfipour S, Leslie FM (2019) Prenatal nicotine sex-dependently alters adolescent dopamine system development. Transl Psychiatry 9:304. 31740669 10.1038/s41398-019-0640-1 PMC6861272

[13] Gao MM, Hu F, Zeng XD, Tang HL, Zhang H, Jiang W, Yan HJ, Shi H, Shu Y, Long YS (2020) Hypothalamic proteome changes in response to nicotine and its withdrawal are potentially associated with alteration in body weight. J Proteomics 214:103633. 31911195 10.1016/j.jprot.2020.103633

[14] Gonçalves J, Leitão RA, Higuera-Matas A, Assis MA, Coria SM, Fontes-Ribeiro C, Ambrosio E, Silva AP (2017) Extended-access methamphetamine self-administration elicits neuroinflammatory response along with blood-brain barrier breakdown. Brain Behav Immun 62:306–317. 28237710 10.1016/j.bbi.2017.02.017

[15] Gordon SL, Leube RE, Cousin MA (2011) Synaptophysin is required for synaptobrevin retrieval during synaptic vesicle endocytosis. J Neurosci 31:14032–14036. 21957264 10.1523/JNEUROSCI.3162-11.2011 PMC3188371

[16] Gupta R, van Dongen J, Fu Y, Abdellaoui A, Tyndale RF, Velagapudi V, Boomsma DI, Korhonen T, Kaprio J, Loukola A, Ollikainen M (2019) Epigenome-wide association study of serum cotinine in current smokers reveals novel genetically driven loci. Clin Epigenetics 11:1. 30611298 10.1186/s13148-018-0606-9 PMC6321663

[17] Hammad HM, Imraish A, Azab B, Best AM, Khader YS, Zihlif M (2019) Associations of CYP2A6 Gene Polymorphism with Smoking Status Among Jordanians: Gender-Related Differences. Curr Drug Metab 20:765–770. 31453782 10.2174/1389200220666190827161112

[18] Hodder SL, Feinberg J, Strathdee SA, Shoptaw S, Altice FL, Ortenzio L, Beyrer C (2021) The opioid crisis and HIV in the USA: deadly synergies. Lancet. 10.1016/S0140-6736(21)00391-3 33617769

[19] Imai Y, Ibata I, Ito D, Ohsawa K, Kohsaka S (1996) A novel gene iba1 in the major histocompatibility complex class III region encoding an EF hand protein expressed in a monocytic lineage. Biochem Biophys Res Commun 224:855–862. 8713135 10.1006/bbrc.1996.1112

[20] Jones HE, Kaltenbach K, Heil SH, Stine SM, Coyle MG, Arria AM, O’Grady KE, Selby P, Martin PR, Fischer G (2010) Neonatal abstinence syndrome after methadone or buprenorphine exposure. N Engl J Med 363:2320–2331. 21142534 10.1056/NEJMoa1005359 PMC3073631

[21] Koizumi T, Kerkhofs D, Mizuno T, Steinbusch HWM, Foulquier S (2019) Vessel-Associated Immune Cells in Cerebrovascular Diseases: From Perivascular Macrophages to Vessel-Associated Microglia. Frontiers in Neuroscience 13:1291. 31866808 10.3389/fnins.2019.01291 PMC6904330

[22] Kopec AM, Smith CJ, Ayre NR, Sweat SC, Bilbo SD (2018) Microglial dopamine receptor elimination defines sex-specific nucleus accumbens development and social behavior in adolescent rats. Nat Commun 9:3769. 30254300 10.1038/s41467-018-06118-z PMC6156594

[23] Kumar M, Rainville JR, Williams K, Lile JA, Hodes GE, Vassoler FM, Turner JR (2021) Sexually dimorphic neuroimmune response to chronic opioid treatment and withdrawal. Neuropharmacology 186:108469. 33485944 10.1016/j.neuropharm.2021.108469 PMC7988821

[24] Martin RE, Green MT, Kinkade JA, Schmidt RR, Willemse TE, Schenk AK, Mao J, Rosenfeld CS (2021) Maternal Oxycodone Treatment Results in Neurobehavioral Disruptions in Mice Offspring. eNeuro 8. 10.1523/ENEURO.0150-21.2021 PMC8354714 34312305

[25] Odegaard KE, Gallegos G, Koul S, Schaal VL, Vellichirammal NN, Guda C, Dutoit AP, Lisco SJ, Yelamanchili SV, Pendyala G (2022) Distinct Synaptic Vesicle Proteomic Signatures Associated with Pre- and Post-Natal Oxycodone-Exposure. Cells 11. 10.3390/cells11111740 PMC9179517 35681434

[26] Odegaard KE, Schaal VL, Clark AR, Koul S, Gowen A, Sankarasubramani J, Xiao P, Guda C, Lisco SJ, Yelamanchili SV, Pendyala G (2020a) Characterization of the intergenerational impact of in utero and postnatal oxycodone exposure. Transl Psychiatry 10:329. 32968044 10.1038/s41398-020-01012-z PMC7511347

[27] Odegaard KE, Schaal VL, Clark AR, Koul S, Sankarasubramanian J, Xia Z, Mellon M, Uberti M, Liu Y, Stothert A, Van Hook M, Wang H, Guda C, Lisco SJ, Pendyala G, Yelamanchili SV (2020b) A Holistic Systems Approach to Characterize the Impact of Pre- and Post-natal Oxycodone Exposure on Neurodevelopment and Behavior. Front Cell Dev Biol 8:619199. 33490084 10.3389/fcell.2020.619199 PMC7817773

[28] Pinho-Ribeiro FA, Verri WA, Chiu IM (2017) Nociceptor Sensory Neuron–Immune Interactions in Pain and Inflammation. Trends in Immunology 38:5–19. 27793571 10.1016/j.it.2016.10.001 PMC5205568

[29] Pittenger ST, Schaal VL, Moore D, Guda RS, Koul S, Yelamanchili SV, Bevins RA, Pendyala G (2018a) MicroRNA cluster miR199a/214 are differentially expressed in female and male rats following nicotine self-administration. Sci Rep 8:17464. 30504847 10.1038/s41598-018-35747-z PMC6269448

[30] Pittenger ST, Schaal VL, Moore D, Guda RS, Koul S, Yelamanchili SV, Bevins RA, Pendyala G (2018b) MicroRNA cluster miR199a/214 are differentially expressed in female and male rats following nicotine self-administration. Scientific Reports 8:17464. 30504847 10.1038/s41598-018-35747-z PMC6269448

[31] Pouryahya P, Birkett W, McR M AD, Louey S, Belhadfa M, Ferdousi S, Imperial K, Nguyen P, Wang A (2020) Oxycodone prescribing in the emergency department during the opioid crisis. Emerg Med Australas 32:996–1000. 32537895 10.1111/1742-6723.13545

[32] Rojas-Rodríguez F, Morantes C, Pinzón A, Barreto GE, Cabezas R, Mariño-Ramírez L, González J (2020) Machine Learning Neuroprotective Strategy Reveals a Unique Set of Parkinson Therapeutic Nicotine Analogs. Open Bioinforma J 13:1–14. 33927788 PMC8081347

[33] Runge K, Cardoso C, de Chevigny A (2020) Dendritic Spine Plasticity: Function and Mechanisms. Front Synaptic Neurosci 12:36. 32982715 10.3389/fnsyn.2020.00036 PMC7484486

[34] Seaton S, Reeves M, McLean S (2007) Oxycodone as a component of multimodal analgesia for lactating mothers after Caesarean section: relationships between maternal plasma, breast milk and neonatal plasma levels. Aust N Z J Obstet Gynaecol 47:181–185. 17550483 10.1111/j.1479-828X.2007.00715.x

[35] Shahjin F, Guda RS, Schaal VL, Odegaard K, Clark A, Gowen A, Xiao P, Lisco SJ, Pendyala G, Yelamanchili SV (2019) Brain-Derived Extracellular Vesicle microRNA Signatures Associated with In Utero and Postnatal Oxycodone Exposure. Cells 9. 10.3390/cells9010021 PMC7016745 31861723

[36] Stevens TR, Krueger SR, Fitzsimonds RM, Picciotto MR (2003) Neuroprotection by nicotine in mouse primary cortical cultures involves activation of calcineurin and L-type calcium channel inactivation. J Neurosci 23:10093–10099. 14602824 10.1523/JNEUROSCI.23-31-10093.2003 PMC6740855

[37] Subramanian J, Michel K, Benoit M, Nedivi E (2019) CPG15/Neuritin Mimics Experience in Selecting Excitatory Synapses for Stabilization by Facilitating PSD95 Recruitment. Cell Rep 28:1584–1595.e1585. 31390571 10.1016/j.celrep.2019.07.012 PMC6740334

[38] van der Put CE, Creemers HE, Hoeve M (2014) Differences between juvenile offenders with and without substance use problems in the prevalence and impact of risk and protective factors for criminal recidivism. Drug Alcohol Depend 134:267–274. 24238911 10.1016/j.drugalcdep.2013.10.012

[39] Wikoff WR, Pendyala G, Siuzdak G, Fox HS (2008) Metabolomic analysis of the cerebrospinal fluid reveals changes in phospholipase expression in the CNS of SIV-infected macaques. J Clin Invest 118:2661–2669. 18521184 10.1172/JCI34138 PMC2398736

[40] Yang L, Yao H, Chen X, Cai Y, Callen S, Buch S (2016) Role of Sigma Receptor in Cocaine-Mediated Induction of Glial Fibrillary Acidic Protein: Implications for HAND. Mol Neurobiol 53:1329–1342. 25631712 10.1007/s12035-015-9094-5 PMC4519438

